# Cardiac Kaposi Sarcoma Complicated by Kaposi Sarcoma Inflammatory Cytokine Syndrome: A Case Report

**DOI:** 10.7759/cureus.76972

**Published:** 2025-01-05

**Authors:** Anna-Maria B Dagher, Jeffrey Lee, Hayley E Cunningham, Julian J Weiss, Michael E Yarrington

**Affiliations:** 1 Internal Medicine, Duke University School of Medicine, Durham, USA; 2 Infectious Diseases, Duke University School of Medicine, Durham, USA

**Keywords:** cardiac kaposi sarcoma, case report, disseminated kaposi sarcoma, hiv aids, immune reconstitution inflammatory syndrome, kshv inflammatory cytokine syndrome (kics), pulmonary kaposi sarcoma

## Abstract

Kaposi sarcoma (KS), caused by human herpesvirus-8 (HHV-8), is among the most common malignancies in people living with HIV. Kaposi sarcoma is an angiogenic endothelial cell neoplasm and an acquired immunodeficiency syndrome (AIDS)-defining illness. Kaposi sarcoma is associated with high mortality when presenting as visceral or disseminated disease or when complicated by Kaposi sarcoma inflammatory cytokine syndrome (KICS), immune reconstitution inflammatory syndrome, or multicentric Castleman disease. We present the case of a 26-year-old male patient found to have AIDS-related KS complicated by cardiac, pulmonary, gastrointestinal, and skin involvement as well as KICS. Notably, the patient’s skin lesions were not identified at the initial hospital presentation. He was treated with doxorubicin for KS and rituximab for KICS but died shortly after KICS diagnosis. We highlight the importance of recognizing KS skin lesions to aid in early diagnosis and of recognizing signs and symptoms of KICS to expedite immunomodulatory therapy initiation.

## Introduction

Acquired immunodeficiency syndrome (AIDS)-related Kaposi sarcoma (KS) is among the most common malignancies in people living with HIV (PLWH) [1]. Gaining public awareness in 1981 during the early AIDS epidemic, KS is an AIDS-defining angiogenic endothelial cell neoplasm caused by human herpesvirus-8 (HHV-8) [2, 3]. Following the development of effective antiretroviral therapy (ART) for HIV in 1996, the incidence of AIDS-related KS in the United States decreased from 47 cases per million people per year in the early 1990s to six cases per million people per year in 2018 [4]. Kaposi sarcoma remains an important clinical entity even in those reliably taking ART, as one-third of AIDS-KS occurs in individuals who have an undetectable HIV viral load [1].

While KS is most recognized for its characteristic cutaneous lesions, which appear dark brown or black on dark skin and purple or red on light skin, KS involves the viscera in up to 50% of cases [5]. Patients who present with localized cutaneous KS have increased survival rates (81%) compared to those with disseminated disease (47%) [4]. Visceral KS is most common in the gastrointestinal and pulmonary systems but can present in virtually any body system, including the heart [2, 6]. Prior to the development of effective ART, autopsies performed on individuals with AIDS-related KS revealed cardiac involvement in 12% to 28% of cases [7].

Development of KS-related syndromes, such as Kaposi sarcoma inflammatory cytokine syndrome (KICS), immune reconstitution inflammatory syndrome (IRIS), and multicentric Castleman disease (MCD), also contribute to poor outcomes [8]. Although each KS-related syndrome is associated with disparate pathophysiology, histopathology, and laboratory findings, all present with symptoms related to systemic inflammation [8]. Both KICS and MCD display sepsis-like symptoms, while KS-IRIS exhibits unmasking of new KS or worsening of known KS following ART initiation [8, 9]. Despite conferring up to 60% mortality, KICS, the focus of this report, remains underrecognized and has no standardized therapy [8, 9].

We report the unique case of a 26-year-old cisgender man with AIDS-related KS complicated by cardiac, pulmonary, and gastrointestinal involvement, as well as KICS. This case report emphasizes the importance of recognizing KS lesions on darker skin tones and maintaining a high degree of clinical suspicion for KICS in at-risk individuals. Additionally, we review the pathophysiology and treatment of KICS, a systemic inflammatory response commonly treated with rituximab and liposomal doxorubicin.

This article was previously presented as a meeting poster at the 2024 American Conference for the Treatment of HIV on May 2, 2024.

## Case presentation

A 26-year-old cisgender male patient with previously diagnosed, untreated HIV presented to the emergency department with nausea, vomiting, sore throat, cough productive of blood-tinged sputum, subjective fever, and chills that started 10 days prior. 

Seven days prior to presentation, he had visited a different emergency department where his vitals were found to be normal. Laboratory findings were significant for hemoglobin of 12.4 g/dL and platelets 92 x 10^9^/L. A chest X-ray showed diffuse pulmonary nodules concerning for miliary tuberculosis, prompting an interferon-gamma release assay and acid-fast bacteria sputum smear, both of which were negative. The patient was diagnosed with influenza A per a positive rapid influenza test and discharged home with amoxicillin given concern for a superimposed atypical bacterial pneumonia. 

**Figure 1:**
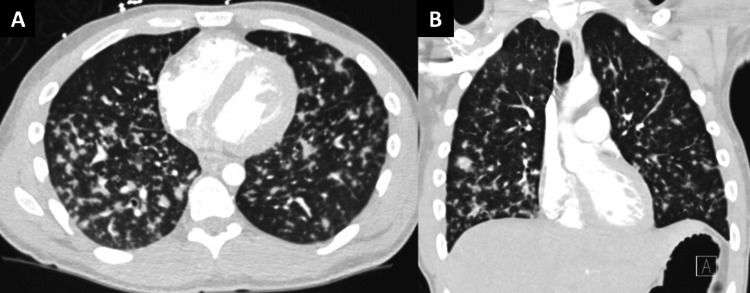
Kaposi sarcoma (KS) pulmonary findings (A) Transverse computed tomography (CT) of the chest showing diffuse bilateral KS pulmonary nodules up to 9 mm in size; (B) Frontal CT of the chest showing KS lesions as described above.

Over the following days, the patient developed worsening night sweats. Seven days following his initial presentation to an outside hospital, the patient presented to our institution’s emergency department. Here, he was found to have tachycardia and tachypnea, with a heart rate of 128 beats/min and a respiratory rate of 30 breaths/min. Temperature, blood pressure, and oxygen saturation were normal. Physical exam at initial presentation was otherwise remarkable for small areas of hyperpigmentation on the upper palate, increased work of breathing, and decreased breath sounds at the bilateral lung bases. The remainder of his physical exam was unremarkable, including a BMI of 23.2 kg/m². Notably, no skin lesions were identified. Initial laboratory results were significant for the following: hemoglobin of 12.1 g/dL, mean corpuscular volume of 73 fl, white blood cell count of 5.7 x 10^9^/L, platelets of 98 x 10^9^/L, C-reactive protein of 7.86 mg/dL, and lactate of 3.9 mmol/L (Table 1). Computed tomography (CT) of the chest showed diffuse bilateral pulmonary nodules up to 9 mm in size with a random distribution pattern (Figure 1). 

**Table 1:** The patient’s initial laboratory results on presentation to the emergency department PCR: polymerase chain reaction; RNA: ribonucleic acid; EIA: enzyme immunoassay; DNA: deoxyribonucleic acid

Laboratory study	Test result	Reference range	Interpretation
Sedimentation rate	115	< 15 mm/hr	High
C-reactive protein	7.86	<= 0.06 mg/dL	High
Lactate	3.9	0.6 – 2.2 mmol/L	High
Magnesium	2.3	1.8 – 2.5 mg/dL	Normal
Comprehensive metabolic panel			
Sodium	133	135 – 145 mmol/L	Low
Potassium	3.7	3.5 – 5.0 mmol/L	Normal
Chloride	96	98 – 108 mmol/L	Low
Carbon dioxide	23	21 – 30 mmol/L	Normal
Blood urea nitrogen	10	7 – 20 mg/dL	Normal
Creatinine	1.0	0.6 – 1.3 mg/dL	Normal
Glucose	141	70 – 140 mg/dL	High
Calcium	8.4	8.7 – 10.2 mg/dL	Low
Aspartate transferase	26	14 – 41 U/L	Normal
Alanine transaminase	14	17 – 63 U/L	Low
Total bilirubin	1.0	0.4 – 1.5 mg/dL	Normal
Alkaline phosphatase	47	24 – 110 U/L	Normal
Albumin	2.7	3.5 – 4.8 g/dL	Low
Total protein	7.0	6.2 – 8.1 g/dL	Normal
Anion gap	14	3 – 12 mmol/L	High
Blood urea nitrogen/Creatinine ratio	10	6 – 27	Normal
Glomerular filtration rate	106	> 60 mL/min/1.73 m^2^	Normal
Complete blood count with differential			
White blood cells	5.7	3.2 – 9.8 x 10^9^/L	Normal
Hemoglobin	12.1	13.7 – 17.3 g/dL	Low
Hematocrit	34.7	39.0 – 49.0%	Low
Platelets	98	150 – 450 x 10^9^/L	Low
Mean corpuscular volume	73	80 – 98 fL	Low
Mean corpuscular hemoglobin	25.5	26.5 – 34.0 pg	Low
Mean corpuscular hemoglobin concentration	34.9	31.5 – 36.3%	Normal
Red blood cell count	4.74	4.37 – 5.74 x 10^12^/L	Normal
Red cell distribution width	16.2	11.5 – 14.5%	High
Nucleated red blood cell count	0.00	0 x 10^9^/L	Normal
Nucleated red blood cell %	0.0	0%	Normal
Neutrophil count	3.1	2.0 – 8.5 x 10^9^/L	Normal
Neutrophil %	55.6	37 – 80%	Normal
Lymphocyte count	1.4	0.6 – 4.2 x 10^9^/L	Normal
Lymphocyte %	24.1	10 – 50%	Normal
Monocyte count	1.1	0 – 0.9 x 10^9^/L	High
Monocyte %	19.6	0 – 12%	High
Eosinophil count	0.00	0 – 0.70 x 10^9^/L	Normal
Eosinophil %	0.0	0 – 7%	Normal
Basophil count	0.01	0 – 0.20 x 10^9^/L	Normal
Basophil %	0.2	0 – 2%	Normal
Immature granulocyte count	0.03	<= 0.06 x 10^9^/L	Normal
Immature granulocyte %	0.5	<= 0.7%	Normal
Venous Blood Gas			
Patient temperature	37.0	36.1 – 37.2°C	Normal
pH	7.51	7.32 – 7.42	High
partial pressure of carbon dioxide (pCO_2_)	30	39 – 55 mmHg	Low
partial pressure of oxygen (pO_2_)	58	30 – 55 mmHg	High
Base excess	2	-3 – 3 mmol/L	Normal
Bicarbonate	24	20 – 28 mmol/L	Normal
Total carbon dioxide (CO_2_)	25	21 – 30 mmol/L	Normal
Hemoglobin	12.2	13.7 – 17.3 g/dL	Low
% oxygen (O_2_) hemoglobin	89.6	60.0 – 85.0%	High
% CO hemoglobin	2.9	<= 2.0%	High
% methemoglobin	0.0	0.4 – 1.5%	Low
Volume % O_2_ venous	15.4	7.0 – 18.0%	Normal
Troponin I	7		
Coronavirus (COVID-19) SARS-CoV-2 rapid test	Not detected	Not detected	Normal
Respiratory virus, extended panel, PCR			
Influenza A RNA	Not detected	Not detected	Normal
Influenza B RNA	Not detected	Not detected	Normal
Respiratory syncytial virus RNA	Not detected	Not detected	Normal
Parainfluenza 1 RNA	Not detected	Not detected	Normal
Parainfluenza 2 RNA	Not detected	Not detected	Normal
Parainfluenza 3 RNA	Not detected	Not detected	Normal
Parainfluenza 4 RNA	Not detected	Not detected	Normal
Human metapneumovirus RNA	Not detected	Not detected	Normal
Adenovirus DNA	Not detected	Not detected	Normal
Human rhinovirus/Enterovirus RNA	Not detected	Not detected	Normal
Fungitell B-D glucan assay	< 31	< 80 pg/mL	Normal
Coccidioides antibody			
Coccidioides complement F	Negative	Negative	Normal
Coccidioides immunodiffusion-IgG	Negative	Negative	Normal
Coccidioides immunodiffusion-IgM	Negative	Negative	Normal
Histoplasma galactomannan antigen	< 0.5	< 0.5 ng/mL	Normal
Histoplasma antigen quantitative EIA	Not detected	Not Detected	Normal
Blastomyces antibody	Negative	Negative	Normal
Cytomegalovirus			
Cytomegalovirus DNA quantitation by PCR	Detected	Not detected	Abnormal
Cytomegalovirus DNA (IU/mL)	< 137	<= 0.0 IU/mL	High
Histoplasma antibody	Negative	Negative	Normal
Cryptococcal antigen	Not detected	Not detected	Normal
Blood culture	No growth detected	No growth detected	Normal
HIV-1 and HIV-2 antibody & antigen			
HIV 1&2 antibody/antigen	Reactive	Non-reactive	Abnormal
HIV antibody	HIV-1 positive	Negative	Abnormal
HIV-1 by PCR, viral load			
HIV-1 8800 500	74,500 copies/mL	<= 0 copies/mL	High
CD4 absolute count	36	400 – 1,400 cells/mm^3^	Low
Mycobacteria culture	No acid-fast bacilli seen or isolated	No acid-fast bacilli seen or isolated	Normal
Methicillin-resistant *Staphylococcus aureus*	Not detected	Not detected	Normal
Hepatitis B surface antibody	Non-reactive	Non-reactive	Normal
*Treponema Pallidum* antibody (syphilis)	Non-reactive	Non-reactive	Normal

The patient was admitted and found to have a CD4 T-cell count of 36 cells/mm³ and an HIV viral load of 74,500 copies/mL. Extensive infectious workup was negative, including respiratory viral panel and bacterial and fungal blood cultures. Additional fungal testing was also negative, including serum beta-D-glucan and galactomannan, serum cryptococcal antigen, urine histoplasma antigen, serum blastomycoses antigen, and sputum fungal cultures. Serum cytomegalovirus was detectable but below the level of quantitation (less than 137 IU/mL), which was not indicative of active disease per infectious disease consultation. 

**Figure 2:**
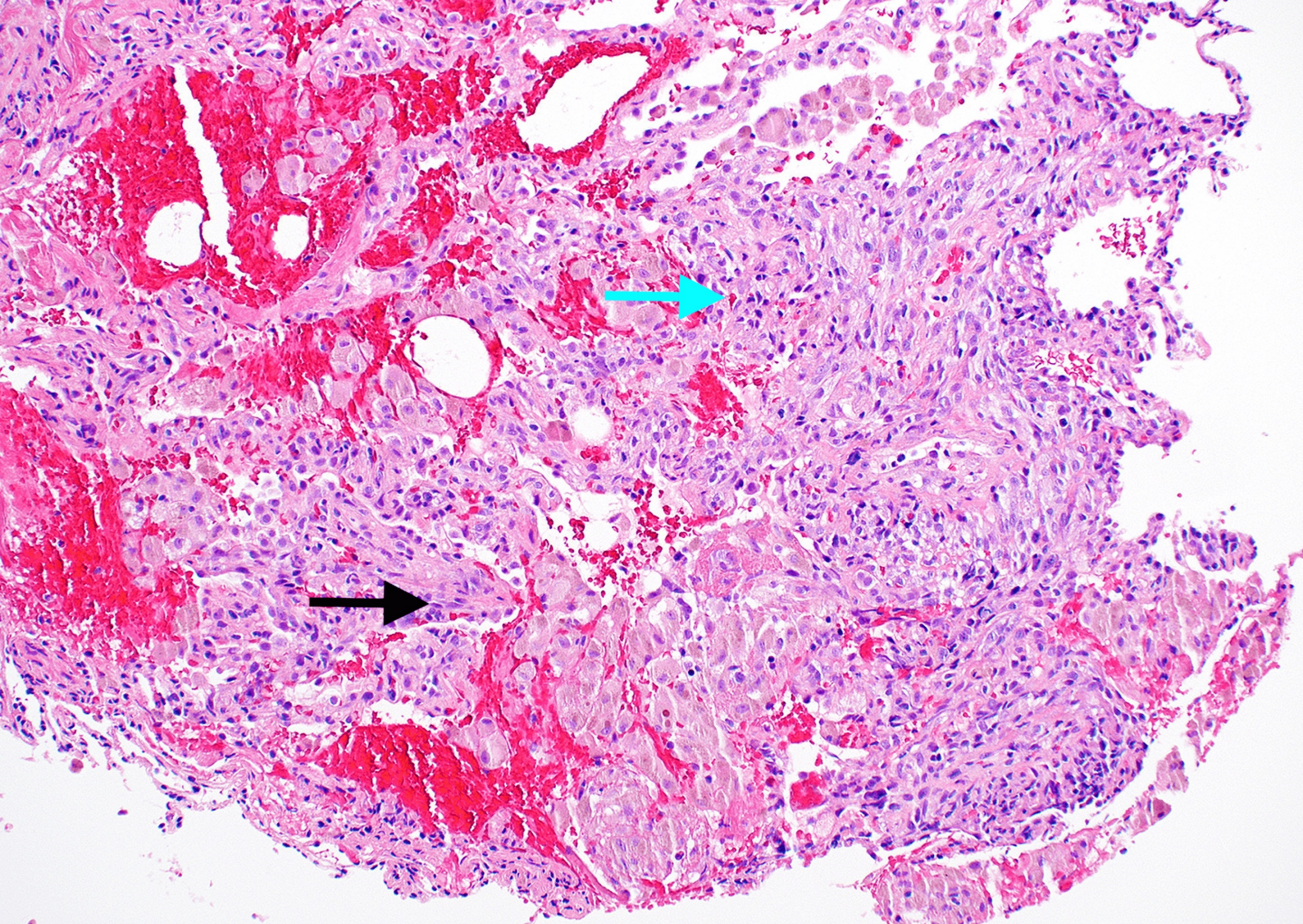
Transbronchial biopsy of the lung revealing Kaposi sarcoma Transbronchial biopsy of the right lung reveals low-grade spindle cell proliferation (black arrow) with occasional vascular “slit-like” spaces, red blood cell extravasation (blue arrow), and rare cytoplasmic eosinophilic globules.

**Figure 3:**
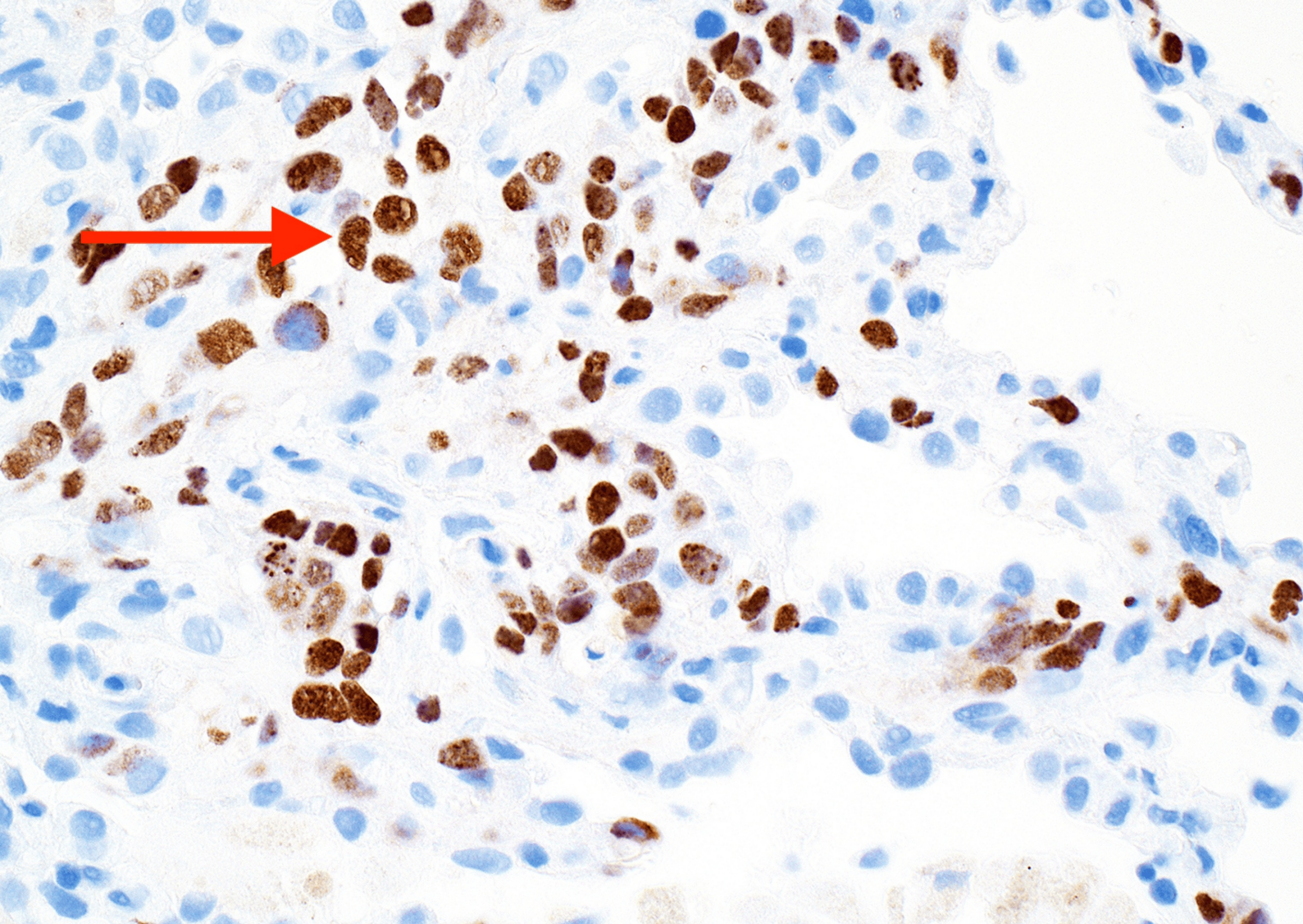
Transbronchial biopsy of the right lung with immunohistochemical staining reveals spindle cell proliferation positive for human herpesvirus-8 (red arrow).

**Figure 4:**
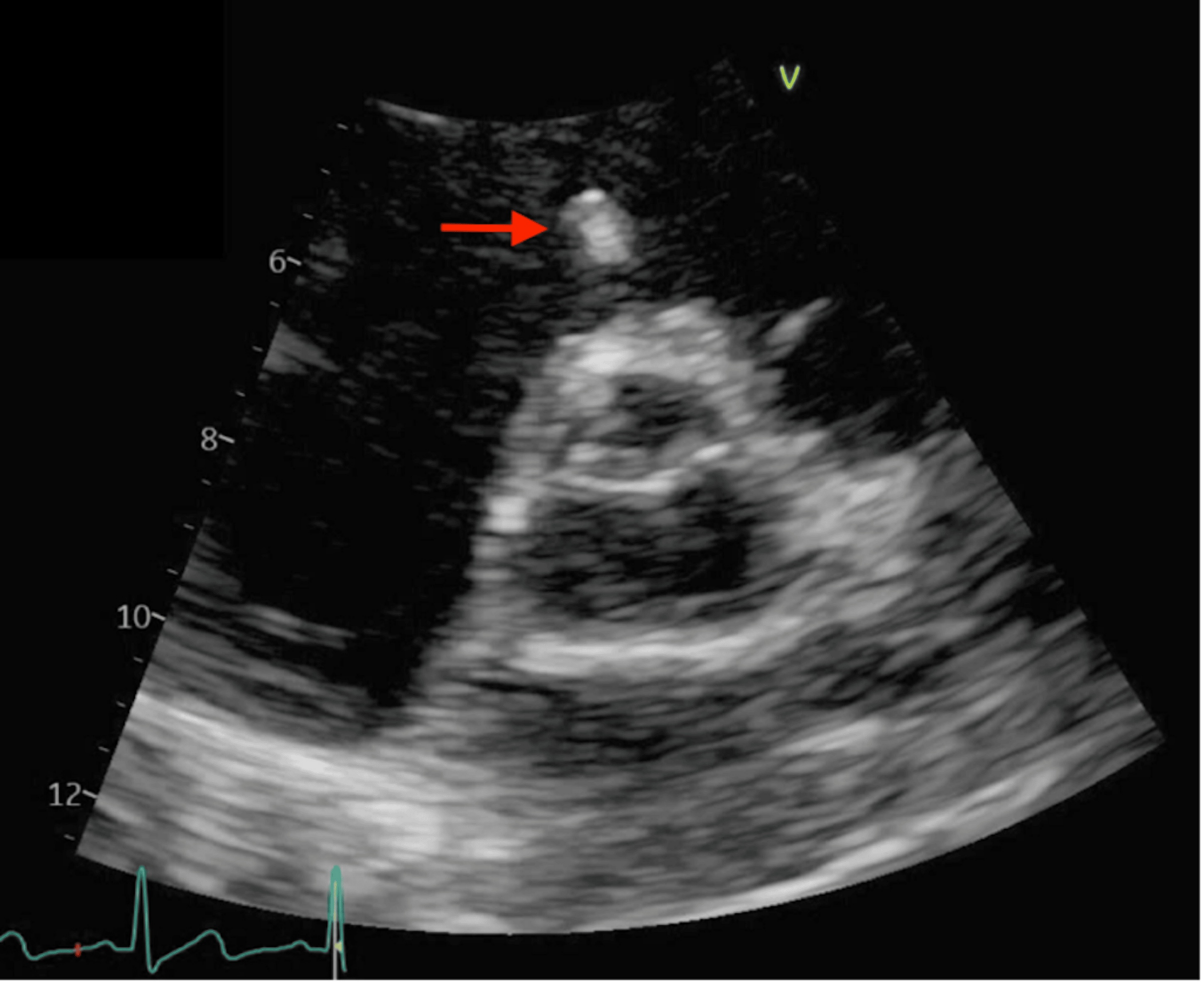
Transesophageal echocardiogram displaying a hyperechoic mobile mass (shown with a red arrow), suggestive of cardiac Kaposi sarcoma.

A transbronchial biopsy revealed KS involving the bilateral lungs (Figures 2-3). A transesophageal echocardiogram showed a hyperechoic mobile mass in the right ventricle concerning for KS involvement (Figure 4). A biopsy of the cardiac mass was not performed because confirmation of cardiac involvement would not have changed management, and a biopsy would have presented significant procedural risk. Similarly, the patient reported bloody mucus in his bowel movements but did not undergo endoscopy or colonoscopy, as confirmation of intestinal involvement would not have changed management.

**Figure 5:**
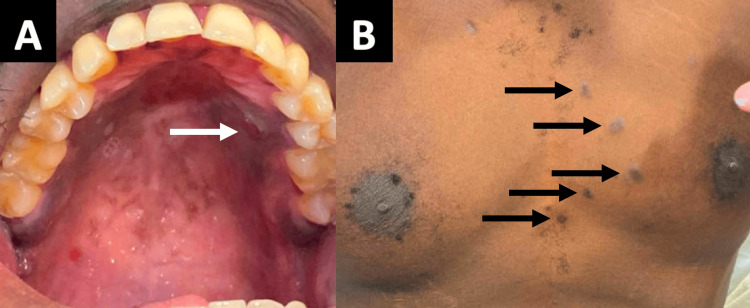
Kaposi sarcoma (KS) lesions on the hard palate and trunk (A) Violaceous-brown KS lesions on the hard palate (representative lesion is shown with a white arrow); (B) Brown-black cutaneous KS lesions on the trunk (representative lesions are shown with black arrows).

Following the pathologic diagnosis of KS from pulmonary tissue, the patient was noted to have a hard palate lesion and multiple lesions on his chest wall consistent with cutaneous KS (Figure 5). No clinician had recognized these as KS lesions prior to this point, likely due to unfamiliarity with the appearance of KS lesions on darker skin tones. The patient had previously noticed the skin lesions but attributed them to the use of hair removal cream. 

During his hospitalization, the patient began KS treatment consisting of chemotherapy with intravenous liposomal doxorubicin 20 mg/m^2^ through a peripherally inserted central catheter every two to three weeks. Total treatment duration was predicted to be six to nine months pending disease response and medication tolerability. He was also started on bictegravir/emtricitabine/tenofovir alafenamide for HIV/AIDS, trimethoprim-sulfamethoxazole for pneumocystis pneumonia prophylaxis, and a multivitamin due to severe protein-calorie malnutrition. 

**Figure 6:**
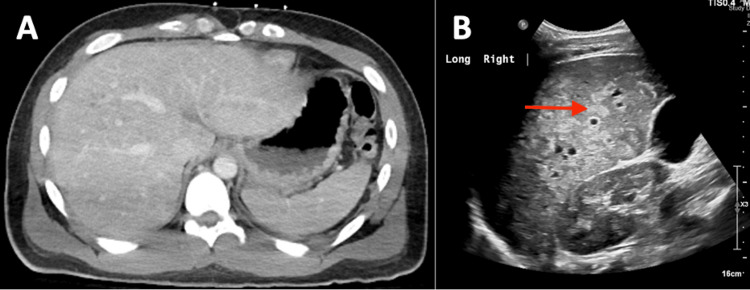
Hepatic Kaposi sarcoma (KS) on abdominal imaging Abdominal imaging revealed numerous subcentimeter lesions scattered throughout the liver representing probable hepatic KS. The lesions are very subtle on computed tomography (a) and more apparent on ultrasound (shown with red arrow) (B).

**Figure 7:**
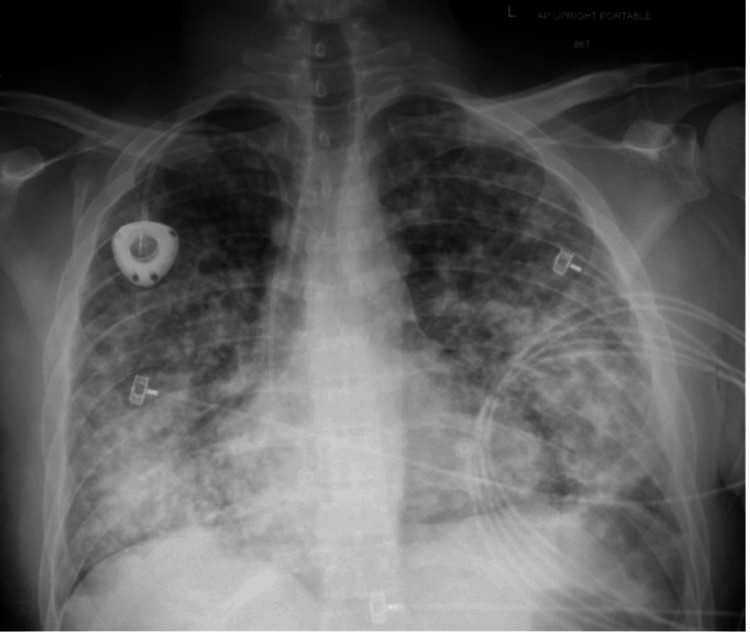
Worsening pulmonary Kaposi sarcoma (KS) on chest X-ray Chest X-ray showing worsening pulmonary nodules indicative of pulmonary KS progression.

For the three months following hospitalization, the patient continued to experience cough and dyspnea on exertion but did see improvements in his skin lesions, which regressed from violaceous papules to dark brown macules. During this time, he was adherent with doxorubicin and ART. Three months after his initial KS diagnosis, the patient was briefly hospitalized for suspected KS-IRIS based on a two-fold increase in his CD4 T-cell count after ART initiation along with elevated HHV-8 antibodies, both of which were suggestive of immune reconstitution. During this hospitalization, he was incidentally found to have diffuse hepatic hypodensities on CT, likely representing KS liver involvement (Figure 6). Additionally, a chest X-ray showed worsening of pulmonary nodules (Figure 7). His symptoms improved with supportive care, and he was discharged five days following admission.

One month later, the patient presented by ambulance to our institution’s emergency department with acute-onset dyspnea, three days of a cough productive of reddish-brown sputum, and fever. While in the ambulance, the patient was febrile at 39.4°C, displayed systolic blood pressure decreasing from 120 mmHg to 109 mmHg and oxygen saturation in the 70s. Prior to arrival, he was given acetaminophen and lactated ringers and placed on a non-rebreather mask at 15 L/min. On arrival to the emergency department, the patient’s vital signs were remarkable for tachycardia and tachypnea, with a heart rate of 109 beats/min and a respiratory rate of 21 breaths/min. His blood pressure was 116/79 mmHg, and oxygen saturation was 95% on the non-rebreather mask. On physical exam, the patient was noted to be in acute distress, although alert and conversant. The exam was otherwise notable for accessory respiratory muscle usage, along with bilateral rhonchi and diminished breath sounds in the lung bases. His hypoxic respiratory failure prompted intubation. The patient was then admitted to the medical intensive care unit, where he was diagnosed with KICS based on his severe, sepsis-like presentation with associated acute respiratory distress syndrome, low CD4 count, and onset outside the timeframe typical for IRIS (less than three months) [8]. He was started on rituximab and discharged 15 days later with supplemental oxygen by nasal cannula (4 L at rest and 6 L to 8 L with ambulation). 

On arrival to oncology follow-up five days after discharge, the patient was hypoxic with an oxygen saturation of 74%-84% on 4 L-7 L of oxygen. Labs were notable for platelets 39 x 10^9^/L and bilirubin trending upwards to 1.6 mg/dL. His aspartate aminotransferase (AST; 26 U/L) and alanine aminotransferase (ALT; 23 U/L) levels were within normal limits. The patient was advised to present to the emergency department, but he ultimately decided against doing so. Upon returning home that day, his respiratory failure worsened, and the patient died. 

## Discussion

The present case demonstrates several features associated with AIDS-KS, including characteristic skin lesions that were not identified during initial clinical encounters. This case also highlights several less common features: KS-IRIS, KICS, and cardiac KS. 

Kaposi sarcoma typically presents with characteristic purple, red, dark brown, or black nodular or macular skin lesions that are often found on the trunk, face, and/or oral mucosa [2, 6]. In the presence of immunocompromising conditions, these cutaneous lesions should prompt high clinical suspicion for KS prior to biopsy confirmation [6]. Skin findings can be purely cutaneous or disseminated to visceral organs, most commonly within the pulmonary and gastrointestinal systems. Less commonly, involvement of the heart, liver, spleen, bone, bone marrow, skeletal muscle, adrenal glands, lymph nodes, pancreas, testes, central nervous system, and kidneys can occur [2, 6]. With widespread ART use, visceral KS involvement is less common [2, 6]. Specifically, from 1996 to 2003, the incidence of visceral disease decreased by 50% [10]. As with the diagnosis of cutaneous KS, the gold standard for the diagnosis of visceral KS is histopathology [1]. However, in the presence of suspected, symptomatic visceral disease, clinicians must weigh the risks and benefits of biopsy. If the risk of biopsy is significant, visceral KS may be visualized using modalities including chest radiography, CT scans, bronchoscopy, or endoscopy [3]. The treatment for visceral or disseminated KS is initiation of ART, plus chemotherapy in severe cases. Doxorubicin and paclitaxel are most often utilized and display similar efficacy [11]. Compared to paclitaxel, doxorubicin is typically preferred as it is associated with lower rates of high-grade toxicity [1, 11]. 

Kaposi sarcoma with minimal cutaneous involvement presents a diagnostic challenge. Differential diagnoses may include bacillary angiomatosis, AIDS-related lymphoma, angiosarcoma, hemangioma, pyogenic granuloma, and dermatofibroma, among others [5]. These skin lesions may appear similar, and a biopsy is necessary for definitive differentiation. For this patient, failure to recognize skin lesions that could have been easily biopsied prompted a more invasive procedure, transbronchial biopsy, for KS diagnosis. Although this patient already had advanced disseminated disease at presentation, such that prompt recognition of skin lesions would not have changed his outcome, recognition of skin lesions could potentially expedite diagnosis and treatment in patients presenting earlier in the disease process. There are many reasons why KS lesions may go unnoticed, including minimal medical trainee exposure to KS lesions on dark skin tones [12]. To increase the timeliness of KS diagnosis, expanding medical education and literature to highlight cutaneous lesions on a variety of skin tones is critical [13]. In an effort to improve recognition of KS on various skin tones, the U.S. Department of Veterans Affairs provides a robust image library of KS lesions [14].

A multitude of inflammatory complications related to KS may occur and are associated with grave prognoses. Many of these complications, including KS-IRIS and KICS, can be difficult to distinguish. Key features are summarized in Table 2. Immune reconstitution inflammatory syndrome is an inflammatory condition that typically occurs within three months of ART initiation due to the restoration of the patient’s immune response [15, 16]. Clinically, IRIS presents as either the unmasking of a new infection or worsening of a known infection and is diagnosed by meeting a certain number of major and/or minor clinical and laboratory criteria [8, 16]. In the case of KS, immune system reconstitution leads to initial HHV-8 suppression with subsequent excessive immune system activation and cytokine storm development [16]. Paradoxically, immune system overactivation eventually leads to lytic reactivation of HHV-8 by mononuclear cells [16, 17]. Viral reactivation then further stimulates an inflammatory response, leading to vascular endothelial growth factor expression and resultant KS tumorigenesis [17]. Our patient was initially diagnosed with IRIS based on: 1) proximity of symptoms to ART initiation (less than three months); 2) fulfillment of two out of three minor criteria for KS-IRIS (two-fold increase in CD4 T-cell count after ART initiation and elevated HHV-8 antibodies); and 3) KS progression [16] as evidenced by the patient’s new hepatic lesions on CT and ultrasound (Figure 6) and worsening pulmonary nodules on chest X-ray (Figure 7). There is no uniform treatment approach for IRIS. Some cases, such as those presenting with mild cutaneous involvement, may reverse with ART alone [3, 15]. Under other circumstances, such as disseminated or visceral KS, the urgent addition of chemotherapy to ART is indicated and can be lifesaving [3, 15]. Prior to his IRIS diagnosis, our patient was already being treated with doxorubicin chemotherapy and ART. Thus, additional therapy was not indicated.

**Table 2:** Comparing the features of KS-IRIS and KICS KS-IRIS: Kaposi sarcoma immune reconstitution inflammatory syndrome; KICS: Kaposi sarcoma inflammatory cytokine syndrome; ART: antiretroviral therapy; KS: Kaposi sarcoma; HHV-8: human herpesvirus-8; IL-6: interleukin-6; IL-10: interleukin-10; MCD: multicentric Castleman disease

	KS-IRIS	KICS
Definition	Inflammatory condition that presents following ART initiation with unmasking of new KS or worsening of known KS due to immune system restoration [8, 16]	Systemic inflammatory syndrome in patients infected with both HIV and HHV-8 with the exclusion of other infectious causes [9, 15]
Timeline	Within three months of ART initiation [8]	No association with ART initiation [8]
Signs and symptoms	KS lesion progression, development of new KS lesions, new or worsening KS organ involvement [3, 16]	Severe symptoms, including fever, fatigue, edema, lymphadenopathy, cachexia, respiratory and gastrointestinal distress, arthralgia, myalgia, altered mental status, and neuropathy [8, 15]
Laboratory findings	Increased serum CD4 count following ART initiation [16], low serum HIV level [8], low HHV-8 viral load [8], hematocrit <30% [15]	CD4 count <100, high serum HIV level, elevated HHV-8 viral load (≥1000 copies/mL), elevated inflammatory markers, anemia, thrombocytopenia, hypoalbuminemia, hyponatremia, elevated IL-6 and IL-10 [8, 9]
Diagnosis	Major criteria plus two or more minor criteria; Major criteria include localized disease, severe inflammatory reaction, unusual inflammation in affected tissue, worsening or new appearance of KS lesions or organ involvement; Minor criteria include increased CD4 count following ART initiation, elevated HHV-8 antibodies, KS remission without therapy and while continuing ART [17]	Clinical signs and symptoms as above along with microscopic exclusion of MCD by lymph node biopsy [8, 9]
Treatment	Treatment depends on clinical presentation: In cases of mild cutaneous involvement, KS-IRIS may reverse on ART alone without specific treatment; In cases of disseminated or visceral disease, urgent addition of chemotherapy (doxorubicin > paclitaxel) to ART is required [3, 15]	Rituximab plus liposomal doxorubicin OR high-dose zidovudine plus valganciclovir [8, 18]
Prognosis	5% to 30% mortality [15]	Up to 60% mortality despite treatment [9]

One month after hospitalization for IRIS, the patient was diagnosed with KICS, a newly described, rare, aggressive inflammatory syndrome associated with AIDS-KS [8]. The syndrome has an unknown pathophysiology but is thought to occur secondary to active HHV-8 replication and associated extensive interleukin (IL) production, specifically IL-6 and IL-10 [8]. Immune system overactivation leads to a cytokine storm, hypotension, systemic inflammation, and respiratory failure [8, 18], as seen in our patient who experienced rapidly progressive acute hypoxic respiratory failure. In contrast to KS-IRIS, KICS can arise more than three months following ART initiation and is associated with greater mortality [8, 15]. Kaposi sarcoma inflammatory cytokine syndrome leads to severe sepsis-like symptoms, including fever, respiratory distress, fatigue, altered mental status, edema, gastrointestinal disturbances, cachexia, arthralgia, and myalgia [8, 15]. Additionally, KICS displays elevated HHV-8 and HIV viral loads in the setting of ongoing inflammatory marker elevation, while KS-IRIS presents with low HHV-8 and HIV viral loads (Table 2) [8, 15]. Additional laboratory findings may include elevated C-reactive protein, anemia, thrombocytopenia, hypoalbuminemia, and hyponatremia [18]. Diagnosis of KICS requires the exclusion of KS-MCD, a lymphoproliferative disorder that presents with relapsing and remitting symptoms similar to those seen in KICS [8, 18]. The patient’s lack of constitutional symptoms between hospitalizations, absence of significant lymphadenopathy and splenomegaly, and CD4 count in the low 100s (rather than greater than 200 cells/uL as typically seen in KS-MCD) made KICS the more likely diagnosis [8, 15]. Our patient did not undergo lymph node biopsy because treatment is the same for both KICS and KS-MCD due to similar disease pathophysiology [8]. This confirmation would not have changed management. Although there is no standard treatment for KICS, immunomodulatory therapy (such as the rituximab utilized for this patient) combined with doxorubicin may lead to clinical improvement [8, 18]. To decrease the dysregulated inflammatory response associated with KICS, rituximab depletes cytokine-releasing B-cells, while liposomal doxorubicin targets KS spindle cells, minimizing their proliferation [15, 18]. Notably, if given alone, rituximab can weaken the immune response, allowing for increased KS replication and spread [8, 15, 18]. In comparison to alternative treatment options, including high-dose zidovudine plus valganciclovir, rituximab and liposomal doxorubicin are associated with lower rates of KICS relapse [15] and are thus generally considered the sole viable therapeutic regimen. Additionally, while being treated for KICS, the patient continued his ART prescription without adjustments. 

Finally, this patient was found to have a right ventricular mass on transesophageal echocardiography (Figure 4), most likely representing cardiac KS. Cardiac involvement is typically the result of disease dissemination [7]. While biopsy can confirm cardiac KS, it is rarely indicated because cardiac involvement in known disseminated KS does not alter treatment [6]. Premortem diagnosis of cardiac KS is rare, as clinical findings are not well-defined and many patients do not display cardiac symptoms [7]. Transthoracic echocardiography has been previously utilized in premortem diagnosis with subsequent confirmation on autopsy. Existing literature notes disease preference for the visceral serous pericardium or subepicardial fat and association with pericardial effusions [19, 20]. Consistent with other reported cases of cardiac KS, the current patient did not display cardiac symptoms, dysfunction, or cardiac-related morbidity/mortality [7]. Nevertheless, cardiac KS is typically associated with a poor prognosis [19]. 

## Conclusions

Although the incidence of AIDS-related KS has decreased significantly with the widespread use of effective ART, the disease remains a clinical concern and can lead to grave outcomes. The current case of KS complicated by cardiac, pulmonary, gastrointestinal, and multiple inflammatory manifestations in a 26-year-old individual highlights the importance of early HIV diagnosis and treatment in young adults. If HIV testing is positive, medical teams must have a plan to ensure patients are not lost to follow-up. All medical providers caring for PLWH should look for and recognize the dermatological manifestations of KS in darker skin tones to aid in early KS diagnosis. Likewise, medical education should include exposure to cutaneous lesions on diverse skin tones. It is also important to recognize the inflammatory signs and symptoms of KS-IRIS and KICS to expedite oncology consultation and initiation of both immunotherapy and chemotherapy. To improve awareness of KS-IRIS and KICS among healthcare professionals, departments of infectious diseases and oncology may consider incorporating formal teaching regarding KS complications into their program structures. Timely treatment of KS and KICS is essential and can be lifesaving. However, more research is needed to define alternative treatment regimens that may lead to improved patient outcomes.

## References

[1] Schneider JW, Dittmer DP (2017). Diagnosis and treatment of Kaposi sarcoma. Am J Clin Dermatol.

[2] Fardin RB, Leite LA, Bezerra LM (2018). Fatal disseminated Kaposi's sarcoma in two patients with human immunodeficiency virus (HIV) infection. Am J Case Rep.

[3] Cesarman E, Damania B, Krown SE, Martin J, Bower M, Whitby D (2019). Kaposi sarcoma. Nat Rev Dis Primers.

[4] Surveillance Research Program, NCI NCI (2024). All cancer sites combined: recent trends in Seer age-adjusted incidence rates, 2000-2021. https://seer.cancer.gov/statistics-network/explorer/.

[5] Sissolak G, Mayaud P (2005). AIDS-related Kaposi's sarcoma: epidemiological, diagnostic, treatment and control aspects in sub-Saharan Africa. Trop Med Int Health.

[6] Lababidi MH, Alhawasli H, Iroegbu N (2015). Kaposi sarcoma can also involve the heart. J Community Hosp Intern Med Perspect.

[7] Barbaro G (2002). Cardiovascular manifestations of HIV infection. Circulation.

[8] Cantos VD, Kalapila AG, Ly Nguyen M, Adamski M, Gunthel CJ (2017). Experience with Kaposi sarcoma herpesvirus inflammatory cytokine syndrome in a large urban HIV clinic in the United States: case series and literature review. Open Forum Infect Dis.

[9] Al-Obaidi A, Mahadevia H, Syed Z, Raza S (2023). A challenging case of Kaposi sarcoma inflammatory cytokine syndrome. Cureus.

[10] Grabar S, Abraham B, Mahamat A, Del Giudice P, Rosenthal E, Costagliola D (2006). Differential impact of combination antiretroviral therapy in preventing Kaposi's sarcoma with and without visceral involvement. J Clin Oncol.

[11] Cianfrocca M, Lee S, Von Roenn J (2010). Randomized trial of paclitaxel versus pegylated liposomal doxorubicin for advanced human immunodeficiency virus-associated Kaposi sarcoma: evidence of symptom palliation from chemotherapy. Cancer.

[12] Kaundinya T, Kundu RV (2021). Diversity of skin images in medical texts: recommendations for student advocacy in medical education. J Med Educ Curric Dev.

[13] Gloster HM Jr, Neal K (2006). Skin cancer in skin of color. J Am Acad Dermatol.

[14] (2025). Kaposi sarcoma/Human herpesvirus 8 (HHV-8). https://www.hiv.va.gov/provider/image-library/ks-hhv-8.asp?thumbs=2.

[15] Dumic I, Radovanovic M, Igandan O (2020). A fatal case of Kaposi sarcoma immune reconstitution syndrome (KS-IRIS) complicated by Kaposi sarcoma inflammatory cytokine syndrome (KICS) or multicentric Castleman disease (MCD): a case report and review. Am J Case Rep.

[16] Abdeljaleel F, Azar J, Ayasa LA, Rabaia D (2024). Kaposi sarcoma-induced immune reconstitution syndrome: a case report. Ann Med Surg (Lond).

[17] Leidner RS, Aboulafia DM (2005). Recrudescent Kaposi's sarcoma after initiation of HAART: a manifestation of immune reconstitution syndrome. AIDS Patient Care STDS.

[18] Karass M, Grossniklaus E, Seoud T, Jain S, Goldstein DA (2017). Kaposi sarcoma inflammatory cytokine syndrome (KICS): a rare but potentially treatable condition. Oncologist.

[19] Rerkpattanapipat P, Wongpraparut N, Jacobs LE, Kotler MN (2000). Cardiac manifestations of acquired immunodeficiency syndrome. Arch Intern Med.

[20] Chyu KY, Birnbaum Y, Naqvi T, Fishbein MC, Siegel RJ (1998). Echocardiographic detection of Kaposi's sarcoma causing cardiac tamponade in a patient with acquired immunodeficiency syndrome. Clin Cardiol.

